# RIP1, RIP3, and MLKL Contribute to Cell Death Caused by Clostridium perfringens Enterotoxin

**DOI:** 10.1128/mBio.02985-19

**Published:** 2019-12-17

**Authors:** Archana Shrestha, Iman Mehdizadeh Gohari, Bruce A. McClane

**Affiliations:** a Department of Microbiology and Molecular Genetics, University of Pittsburgh School of Medicine, Pittsburgh, Pennsylvania, USA; University of Oklahoma Health Sciences Center

**Keywords:** *Clostridium perfringens* enterotoxin, apoptosis, necroptosis, RIP1 kinase, RIP3 kinase, MLKL, calpain, *Clostridium perfringens*, RIP1, RIP3, enterotoxin

## Abstract

Clostridium perfringens type F strains cause gastrointestinal disease when they produce a pore-forming toxin named C. perfringens enterotoxin (CPE). In human enterocyte-like Caco-2 cells, low CPE concentrations cause caspase-3-dependent apoptosis, while high CPE concentrations cause necrosis. Since necrosis or apoptosis sometimes involves receptor-interacting serine/threonine-protein kinase-1 or 3 (RIP1 or RIP3), this study examined whether those kinases are important for CPE-induced apoptosis or necrosis. Highly specific RIP1 or RIP3 inhibitors reduced both CPE-induced apoptosis and necrosis in Caco-2 cells. Those findings suggested that the form of necrosis induced by treating Caco-2 cells with high CPE concentrations involves necroptosis, which was confirmed when high, but not low, CPE concentrations were shown to induce oligomerization of mixed-lineage kinase domain-like pseudokinase (MLKL), a key late step in necroptosis. Furthermore, an MLKL oligomerization inhibitor reduced cell death caused by high, but not low, CPE concentrations. Supporting RIP1 and RIP3 involvement in CPE-induced necroptosis, inhibitors of those kinases also reduced MLKL oligomerization during treatment with high CPE concentrations. Calpain inhibitors similarly blocked MLKL oligomerization induced by high CPE concentrations, implicating calpain activation as a key intermediate in initiating CPE-induced necroptosis. In two other CPE-sensitive cell lines, i.e., Vero cells and human enterocyte-like T84 cells, low CPE concentrations also caused primarily apoptosis/late apoptosis, while high CPE concentrations mainly induced necroptosis. Collectively, these results establish that high, but not low, CPE concentrations cause necroptosis and suggest that RIP1, RIP3, MLKL, or calpain inhibitors can be explored as potential therapeutics against CPE effects *in vivo*.

## Abstract (precis)

C. perfringens type F strains are a common cause of food poisoning and antibiotic-associated diarrhea. Type F strain virulence requires production of C. perfringens enterotoxin (CPE). In Caco-2 cells, high CPE concentrations cause necrosis while low enterotoxin concentrations induce apoptosis. The current study determined that receptor-interacting serine/threonine-protein kinases 1 and 3 are involved in both CPE-induced apoptosis and necrosis in Caco-2 cells, while mixed-lineage kinase domain-like pseudokinase (MLKL) oligomerization is involved in CPE-induced necrosis, thereby indicating that this form of CPE-induced cell death involves necroptosis. High CPE concentrations also caused necroptosis in T84 and Vero cells. Calpain activation was identified as a key intermediate for CPE-induced necroptosis. These results suggest inhibitors of RIP1, RIP3, MLKL oligomerization, or calpain are useful therapeutics against CPE-mediated diseases.

## INTRODUCTION

Clostridium perfringens enterotoxin (CPE) is produced only during the sporulation of C. perfringens (1). CPE is a 35-kDa single polypeptide that has a unique amino acid sequence, except for limited homology, of unknown significance, with a nonneurotoxic protein made by Clostridium botulinum (2). Structurally, CPE consists of two domains and belongs to the aerolysin family of pore-forming toxins (3, 4). The C-terminal domain of CPE mediates receptor binding (5, 6), while the N-terminal domain of this toxin is involved in oligomerization and pore formation (7, 8).

CPE production is required for the enteric virulence of C. perfringens type F strains (9), which were formerly known as CPE-positive C. perfringens type A strains prior to the recent revision of the C. perfringens isolate classification system (10). Type F strains are responsible for C. perfringens type F food poisoning (formerly known as C. perfringens type A food poisoning), which is the 2nd most common bacterial foodborne illness in the United States, where about 1 million cases/year occur (11). This food poisoning is typically self-limiting but can be fatal in the elderly or people with pre-existing fecal impaction or severe constipation due to side effects of medications taken for psychiatric illnesses (12, 13). Type F strains also cause 5 to 10% of nonfoodborne human gastrointestinal diseases, including sporadic diarrhea or antibiotic-associated diarrhea (14).

The cellular action of CPE begins when this toxin binds to host cell receptors, which include certain members of the claudin family of tight junction proteins (15). This binding interaction results in formation of an ∼90-kDa small complex that is comprised of CPE, a claudin receptor, and a nonreceptor claudin (16). Several (approximately six) small complexes then oligomerize to form an ∼425- to 500-kDa prepore complex on the surface of host cells (16). Beta hairpin loops are extended from each CPE molecule present in the prepore to create a beta-barrel that inserts into the host cell membrane and forms a pore (8).

The pore formed by CPE is highly permeable to small molecules, particularly cations such as Ca^2+^ (17). In enterocyte-like Caco-2 cells treated with relatively low (1 μg/ml) CPE concentrations, calcium influx is modest and results in limited calpain activation that causes a classical apoptosis involving mitochondrial membrane depolarization, cytochrome *c* release, and caspase-3 activation (17, 18). Importantly, this CPE-induced apoptotic cell death is caspase-3 dependent, since specific inhibitors of this caspase reduce the cell death caused by treatment with 1 μg/ml CPE (17, 18). In contrast, when Caco-2 cells are treated with higher (but still pathophysiologic [19]) CPE concentrations, a massive calcium influx occurs that triggers strong calpain activation and causes cells to die from a form of necrosis initially referred to as oncosis (18). Caspase-3 or -1 inhibitors do not affect this form of CPE-induced cell death, but transient protection is afforded by the presence of glycine, a membrane stabilizer (18). Cell death mechanisms appear to be important for understanding CPE-induced enteric disease, since only recombinant CPE variants that are cytotoxic for cultured cells *in vitro* are capable of causing intestinal damage and intestinal fluid accumulation in animal models (20).

Since the original research on CPE-induced Caco-2 cell death was reported ∼15 years ago (17, 18), considerable progress has been achieved toward understanding the molecular mechanisms behind mammalian cell death (21). Of particular note, additional forms of cell death have now been identified and the pathways behind many cell death mechanisms have been further elucidated. For example, multiple forms of apoptosis and necrosis are now recognized, including a form of programmed necrosis named necroptosis (22). Similarly, a number of additional host proteins mediating cell death have been identified. Among these are receptor-interacting serine/threonine-protein (RIP) kinase family members RIP1 and RIP3, which are sometimes involved in necrosis or apoptosis. As an example, when RIP1 and RIP3 are phosphorylated in response to appropriate cell death stimuli, they can interact with other proteins to form the necrosome (21, 22). Necroptosis then results when the necrosome phosphorylates mixed-lineage kinase domain-like pseudokinase (MLKL) to induce formation of a large MLKL oligomer, which is a necroptosis effector (21, 22).

The possible contributions of RIP1, RIP3, and MLKL to CPE-induced cell death have not yet been investigated. If CPE-induced cell death does involve RIP1, RIP3, or MLKL, these proteins could represent potential therapeutic targets to reduce CPE activity *in vivo*, since highly specific inhibitors are available against each of these host proteins (23–25). Considering those two points, the current study evaluated the involvement of these cell death pathway proteins in CPE-induced cell death.

## RESULTS

### Caco-2 cells produce RIP1, RIP3, and MLKL.

**FIG 1:**
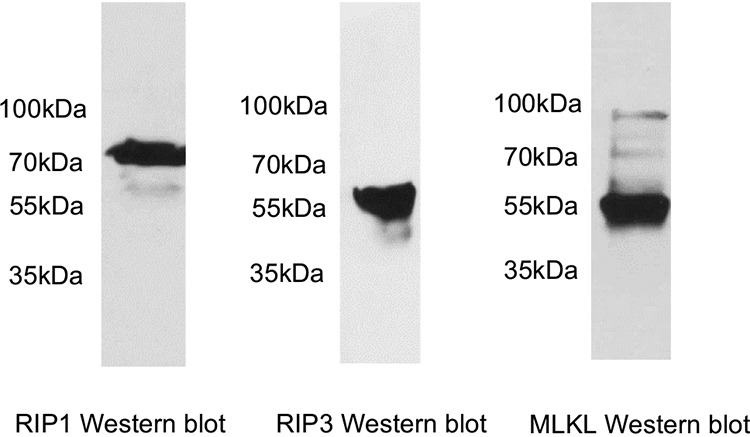
Production of RIP1, RIP3, and MLKL by Caco-2 cells. Confluent Caco-2 cell cultures were lysed with RIPA buffer. Lysates were electrophoresed, and separated proteins were then Western blotted with an antibody against RIP1 (3493S; Cell Signaling Technology), RIP3 (NBP2-24588; Novus), or MLKL (14993S; Cell Signaling Technology). Using the RIP1 antibody, an ∼76-kDa immunoreactive band was detected in Caco-2 lysates, matching the expected size of RIP1. With the RIP3 antibody, an ∼57-kDa immunoreactive band was detected, matching the expected size of RIP3. Using the MLKL antibody, an ∼54-kDa immunoreactive band was detected, matching the expected size of MLKL. Similar results were obtained if the Western blot was repeated (not shown) with RIP1 antibody (610458; BD Biosciences), RIP3 antibody (13526S; Cell Signaling Technology), or MLKL antibody (GTX 107538; GeneTex).

Before assessing whether RIP1, RIP3, and MLKL inhibitors are involved in CPE-induced death of Caco-2 cells, it was necessary to demonstrate that these cells produce all three of these host proteins. Western blot analysis of Caco-2 cell lysates using a validated RIP1 antibody detected the presence of an immunoreactive ∼76-kDa protein (Fig. 1, left), matching the expected size of RIP1. Similarly, Western blot analyses of those lysates using validated RIP3 or MLKL antibodies demonstrated the presence of immunoreactive proteins of ∼57 kDa (Fig. 1, middle) or ∼54 kDa (Fig. 1, right), matching the expected size of RIP3 or MLKL, respectively. Similar Western blot results were obtained using a second antibody against each of these three host proteins (data not shown).

### Effects of RIP1 inhibitors on CPE-induced cell death in Caco-2 cells.

**FIG 2:**
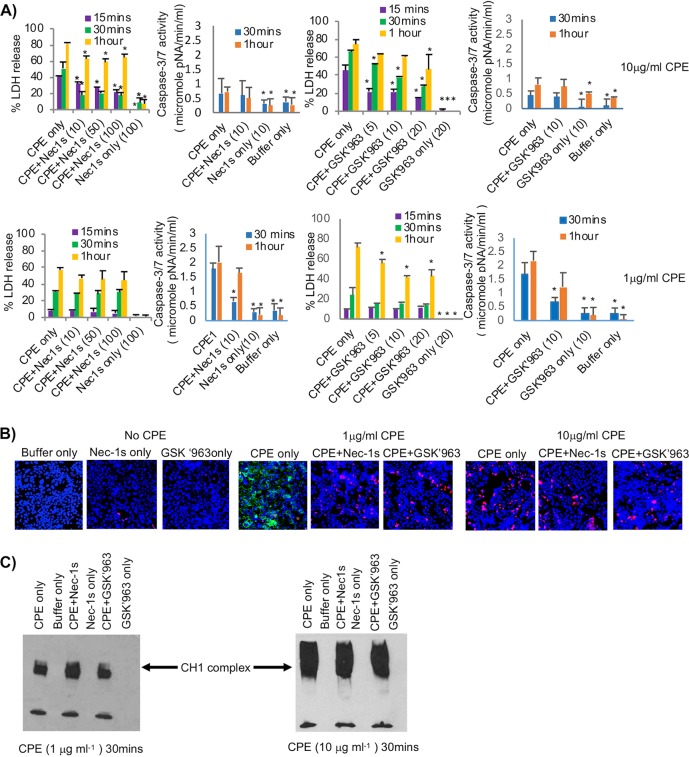
Effects of RIP1 inhibitors on CPE-induced cell death. (A) LDH release or caspase-3 activation. Caco-2 cells were pretreated with or without an RIP1 inhibitor (Nec-1s or GSK′963) at concentrations indicated in parentheses and then treated for the indicated times at 37°C with HBSS or HBSS containing 10 μg ml^−1^ (top row) or 1 μg ml^−1^ (bottom row) of CPE, along with the same RIP1 inhibitor concentration used, if any, for preincubation. For each row, the 1st and 3rd panels show LDH release, while the 2nd and 4th panels depict caspase-3 activation. Results shown are the means from 3 repetitions. Error bars represent the standard errors of the means (SEM). An asterisk indicates significantly different (*P* ≤ 0.05) values from CPE alone (no inhibitor). (B) Fluorescence microscopy. Caco-2 cells pretreated with HBSS with or without a 10 μM concentration of Nec-1s or GSK′963 were challenged for 30 min at 37°C with HBSS that did not contain CPE (left) or contained 1 μg ml^−1^ (middle) or 10 μg ml^−1^ (right) CPE and (if used for pretreatment) 10 μM concentrations of Nec-1s or GSK′963. Treated cultures were then stained with an apoptotic/necrotic/healthy cell detection kit, where green cells are apoptotic (due to annexin V staining), red cells are necrotic (due to EthD-III staining), and total cells are blue (due to Hoechst 33342 staining). For each treatment, stained cells in at least 3 fields were quantified for each of 3 repetitions. Representative fields are shown. (C) Western blot analysis of CPE pore complex formation. Caco-2 cells pretreated with HBSS that did or did not contain a 10 μM concentration of Nec-1s or GSK′963 and then were treated for 30 min (Fig. 2C) or 1 h (not shown) at 37°C with HBSS that contained 1 μg ml^−1^ (left) or 10 μg ml^−1^ (right) CPE plus the same RIP1 inhibitor concentration, if any, used for pretreatment. Cell lysates were subjected to CPE Western blotting. Representative results from 3 repetitions are shown.

We previously reported (17, 18) that Caco-2 cells treated with a low (1 μg/ml) CPE concentration develop caspase-3-mediated apoptosis, while treatment of those cells with a higher, but still pathophysiologic (19), 10 μg/ml CPE concentration causes a form of necrosis then referred to as oncosis. The current study confirmed those previous observations by demonstrating that Caco-2 cells treated with 10 μg/ml enterotoxin developed substantial (≥50%) early (within 30 min of treatment) release of lactate dehydrogenase (LDH), which is indicative of necrosis (Fig. 2A, top row). In contrast, treatment of Caco-2 cells with 1 μg/ml CPE did not cause similarly high levels of LDH release until >30 min of treatment (Fig. 2A, bottom row), consistent with the development of membrane permeability alterations during late apoptosis (26). In addition and in agreement with previous studies (17, 18) showing that low CPE concentrations induce caspase-3-mediated apoptosis in Caco-2 cells, treatment of these cells with 1 μg/ml enterotoxin caused substantial caspase-3 activation within 30 min, while the 10 μg/ml dose of CPE induced only a slight caspase-3 activation above background levels, even when Caco-2 cells were treated for 1 h with this high toxin concentration (Fig. 2A, top and bottom). The levels of caspase-3 activation caused by 1 μg/ml CPE in Fig. 2A were similar to those reported previously (18), and the amount of caspase-3 activation observed using the low CPE concentration also matched the caspase-3 activation levels induced by staurosporine, a positive control for caspase-3 activation, in that previous study.

This study next pursued its first major goal, i.e., to determine if the presence of RIP1 kinase activity inhibitors impacts the development of CPE-mediated cell death in Caco-2 cells. For this purpose, caspase-3 activation and substantial early LDH release were assessed as biochemical indicators of apoptosis or necrosis, respectively. Even the lowest (10 μM) tested concentration of the RIP1 inhibitor Nec-1s (24, 27) significantly inhibited the substantial early LDH release from Caco-2 cells caused by treatment with 10 μg/ml CPE (Fig. 2A, top row). For example, by 30 min of toxin treatment, the presence of 10 μM Nec-1s caused a 69.5% reduction in the LDH release induced by treatment with this high CPE concentration. Furthermore, significant Nec-1s-mediated protection from high CPE concentration-induced LDH release continued for up to 60 min (Fig. 2A). However, at no time point did the presence of even a high (100 μM) concentration of Nec-1s significantly protect Caco-2 cells from the more delayed LDH release induced by 1 μg/ml CPE (Fig. 2A).

With respect to the caspase-3 activation necessary for CPE-induced apoptosis (18), Nec-1s caused a significant 62.8% reduction in the strong caspase-3 activation induced by 30 min of treatment with 1 μg/ml CPE (Fig. 2A, bottom row). However, this protection was transient, becoming statistically insignificant by 60 min of CPE treatment (Fig. 2A, bottom row). By comparison, at either 30 or 60 min, this RIP1 inhibitor had no significant protective effect on the minimal caspase-3 activation caused by 10 μg/ml CPE (Fig. 2A, top row).

Although Nec-1s is a highly specific RIP1 kinase inhibitor, the effects of a second specific RIP1 kinase inhibitor, named GSK′963 (28), on CPE-induced cell death were also assessed to further reduce the possibility of off-target drug inhibition effects producing the Nec-1s results shown in Fig. 2A. Even a 5 μM concentration of this second RIP1 inhibitor significantly reduced the rapid and substantial release of LDH caused by treatment for 15 or 30 min with 10 μg/ml CPE (Fig. 2A, top row). For example, the presence of a 5 μM concentration of GSK′963 caused a 51.5% reduction in LDH release after a 15-min treatment with this high CPE concentration. In contrast, using the lower 1 μg/ml CPE concentration, the tested concentrations of GSK′963 did not significantly affect early (i.e., 15- or 30-min treatment) LDH release from Caco-2 cells (Fig. 2A, bottom row). Inversely, the presence of 10 μM GSK′963 caused a significant 60.9% reduction in the strong caspase-3 activation cells caused by 30 min of treatment of Caco-2 cells with 1 μg/ml CPE (Fig. 2A, bottom row). However, this inhibitor did not significantly affect the small amount of caspase-3 activation in Caco-2 cells that is induced by treatment with 10 μg/ml toxin (Fig. 2A, top row). Finally, in control experiments, neither RIP1 inhibitor alone (no CPE present) induced significant LDH release or caspase-3 activation above background (Fig. 2A).

The biochemical assay results shown in Fig. 2A indicated that, for Caco-2 cells, RIP1 inhibitors can significantly impact the substantial early release of LDH (a marker for necrosis) that is induced by treatment with high CPE concentrations or the strong early caspase-3 activation (a marker for apoptosis) that is caused by treatment using low CPE concentrations. To confirm that the RIP1 inhibitors were specifically affecting those two CPE-induced cell death pathways, fluorescence microscopy was performed to evaluate the effects of RIP1 inhibitors on the presence of apoptotic or necrotic cells in CPE-treated Caco-2 cell cultures. Those imaging results (Fig. 2B) were consistent with the biochemical assay results shown in Fig. 2A or previously (17, 18). Specifically, in the presence or absence of RIP1 inhibitors without CPE, there was little or no staining of Caco-2 cells with annexin V (which is indicative of apoptosis) or ethidium homodimer III (EthD-III; which is impermeant to live or apoptotic cells but stains necrotic cells). However, in the absence of RIP1 inhibitors, treatment of Caco-2 cells with 1 μg/ml CPE induced apoptosis, while treatment with 10 μg/ml toxin caused necrotic death. For example, the 1 μg/ml concentration of CPE induced 35.6% ± 13.7% of Caco-2 cells to stain with annexin V (green color) after a 30-min treatment. In contrast, no annexin V staining was observed in Caco-2 cells treated for 15 min (not shown) or 30 min (Fig. 2B) with 10 μg/ml CPE. Instead, 17.8% ± 5.1% of Caco-2 cells treated for 30 min with this high CPE dose showed red staining with EthD-III.

The effects of RIP1 inhibitors on the apoptosis induced by treating Caco-2 cells with 1 μg/ml CPE then were investigated using the same fluorescence microscopy-based assay (Fig. 2B). Consistent with results shown in Fig. 2A, the presence of Nec-1s or GSK′963 eliminated annexin V (green) staining after either a 15-min (not shown) or 30-min (Fig. 2B) treatment with this low CPE dose. Instead, a small number (by 30 min, 8.0% ± 0.5% for Nec-1s or 9.0 ± 2.9% for GSK′963) of cells in those cultures stained with EthD-III (red), indicative of necrosis. In the absence of CPE, neither inhibitor caused any increase in annexin V or EthD-III staining above the low background levels (Fig. 2B). Also consistent with results shown in Fig. 2A, no annexin V (green) staining for apoptosis was observed in Caco-2 cells treated for 15 min (not shown) or 30 min (Fig. 2B) with 10 μg/ml CPE in the presence of Nec-1s or GSK′963. Furthermore, both RIP1 inhibitors significantly (*P* ≤ 0.05) decreased the number of CPE-treated necrotic cells staining red with EthD-III after treatment of these cells for 30 min with the high CPE concentration. For example, with this CPE treatment condition, Nec-1s or GSK′963 reduced the number of necrotic cells by 52.0% ± 23.4% or 44.5% ± 12.9%, respectively.

CPE-induced cytotoxicity involves formation of a large CPE pore complex, named CH-1, in sensitive cells (7, 8). If, in the experiments shown in Fig. 2A and B, Nec-1s and GSK′963 were protecting Caco-2 cells from CPE-induced apoptosis or necrosis because RIP1 activation is a consequence of CPE pore formation, these RIP1 inhibitors should be acting after CH-1 pore formation has occurred. Alternatively, Nec-1s or GSK′963 might protect against CPE by somehow indirectly affecting CH-1 formation levels. To discriminate between those possibilities, Western blot analysis was performed using Caco-2 cells treated with 1 or 10 μg/ml CPE in the presence or absence of Nec-1s or GSK′963. For cells treated with either 1 or 10 μg/ml CPE, CPE Western blots did not reveal any inhibitory effects of Nec-1s or GSK′963 on CH-1 pore levels in Caco-2 cells after a 30 (Fig. 2C)- or 60 (not shown)-min CPE treatment.

### Effects of RIP3 inhibitors on CPE-induced cell death in Caco-2 cells.

**FIG 3:**
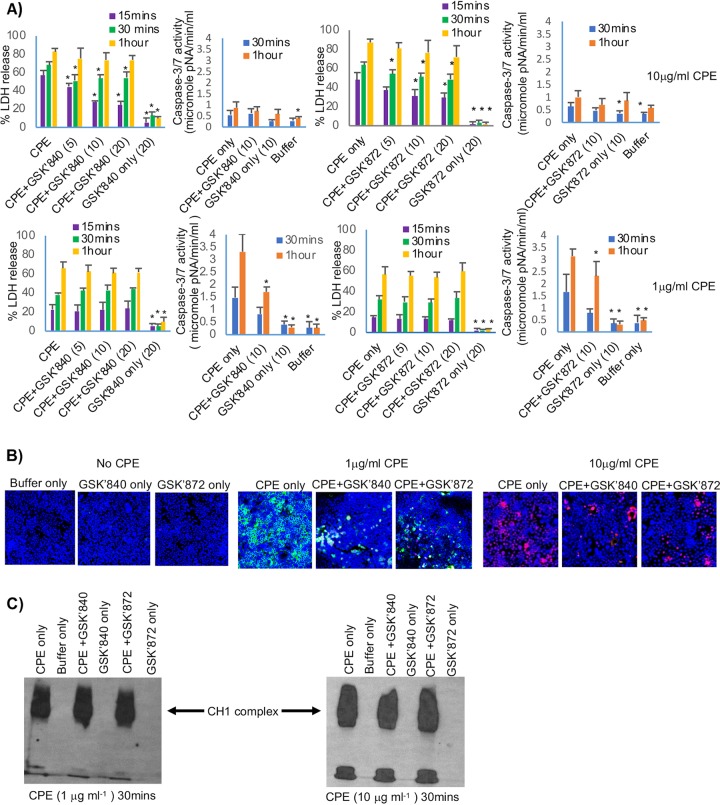
Effects of RIP3 inhibitors on CPE-induced cell death. (A) LDH release or caspase-3 activation. Caco-2 cells were pretreated with HBSS that did or did not contain the RIP3 inhibitor GSK′840 or GSK′872 at the concentrations shown in parentheses and then treated at 37°C for the indicated times with HBSS or HBSS containing 10 μg ml^−1^ (top row) or 1 μg ml^−1^ (bottom row) CPE, along with the same concentration of RIP3 inhibitor, if any, used for preincubation. For each row, the 1st and 3rd panels show LDH release, while the 2nd and 4th panels depict caspase-3 activation. Shown are the means from 3 repetitions; error bars represent SEM. An asterisk indicates significantly different (*P* ≤ 0.05) values from CPE alone (no inhibitor). (B) Fluorescence microscopy. Caco-2 cells were pretreated with HBSS that did or did not contain a 10 μM concentration of GSK′840 or GSK′872 and then were challenged for 30 min at 37°C with HBSS without CPE (left) or containing 1 μg ml^−1^ (middle) or 10 μg ml^−1^ (right) CPE and (if used for pretreatment) a 10 μM concentration of GSK′840 or GSK′872. Treated cultures were stained with an apoptotic/necrotic/healthy cell detection kit, where green cells are apoptotic (due to annexin V staining), red cells are necrotic (due to EthD-III staining), and total cells are blue (due to Hoechst 33342 staining). For each treatment, stained cells in at least 3 fields were quantified for each of 3 repetitions. Representative fields are shown. (C) Western blot analysis of CPE pore complex formation. Caco-2 cells were pretreated with a 10 μM concentration of GSK′840 or GSK′872 before treatment for 30 min (Fig. 3C) or 1 h (not shown) at 37°C with HBSS containing 1 μg ml^−1^ (left) or 10 μg ml^−1^ (right) CPE plus the same RIP3 inhibitor concentration, if any, used for pretreatment. Cell lysates were subjected to CPE Western blotting. Representative results of three repetitions are shown.

Results shown in Fig. 2 indicated that RIP1 is involved in both the necrosis caused by high CPE concentrations and the apoptosis caused by low CPE concentrations. Since RIP3 is also sometimes involved in necrosis or apoptosis (21, 29–32), similar experiments were repeated using inhibitors of RIP3 kinase activity to evaluate if this kinase is also involved in CPE-induced cell death. When present, the GSK′840 RIP3 inhibitor significantly reduced the rapid and substantial LDH release caused by a 15-min (not shown) or 30-min (Fig. 3A, top) treatment with 10 μg/ml CPE. For example, the 10 μM dose of this inhibitor reduced by 51.2% the LDH release caused by a 15-min treatment with this high CPE dose. In contrast, even the 20 μM concentration of this RIP3 inhibitor did not significantly protect Caco-2 cells from the slower-developing LDH release caused by treatment with 1 μg/ml CPE (Fig. 3A, bottom). Inversely, GSK′840 caused a significant 46.3% reduction in the caspase-3 activation induced by 60 min of treatment with a 1 μg/ml dose of CPE (Fig. 3A, bottom). In contrast, this RIP3 inhibitor had no significant effect on the small amount of caspase-3 activation present in Caco-2 cells treated with 10 μg/ml CPE (Fig. 3A, top).

Although GSK′840 is a highly specific RIP3 inhibitor, a second RIP3 kinase inhibitor, named GSK′872 (25), was also used to further reduce the possibility of off-target drug effects explaining the inhibition of CPE-induced cell death observed in the experiments shown in Fig. 3A and B. At both the 15- and 30-min toxin treatment times, the 10 or 20 μM concentration of this RIP3 inhibitor significantly decreased the substantial early release of LDH caused by challenge with this high toxin concentration (Fig. 3A, top). For example, 10 μM GSK′872 caused a 36.6% reduction in the LDH release caused by a 15-min treatment with 10 μg/ml CPE. However, even a 20 μM concentration of GSK′872 did not affect the delayed release of LDH from Caco-2 cells that is caused by a low (1 μg/ml) CPE dose (Fig. 3A, bottom).

In contrast, GSK′872 (10 μM) caused a significant 26.4% reduction in caspase-3 activation following a 60-min treatment of Caco-2 cells with 1 μg/ml CPE (Fig. 3A, bottom). However, this inhibitor did not affect the very small amount of caspase-3 activation that is induced in these cells by treatment with 10 μg/ml CPE (Fig. 3A, top). In control experiments, neither RIP3 inhibitor alone (no CPE) induced significant LDH release or caspase-3 activation above background levels (Fig. 3A).

Results shown in Fig. 3A indicated that, in Caco-2 cells, RIP3 inhibitors can significantly reduce the substantial early release of LDH (a marker for necrosis) induced by a high CPE dose or the caspase-3 activation (a marker for apoptosis) caused by a low CPE dose. To confirm those conclusions, fluorescence microscopy staining was performed to detect whether those RIP3 inhibitors affect the presence of apoptotic or necrotic cells in CPE-treated Caco-2 cell cultures. Using this microscopy-based assay, the results obtained were consistent with results shown in Fig. 3A. Specifically, the presence of either 10 μM GSK′840 or GSK′872 significantly (*P* ≤ 0.05) reduced annexin V staining (apoptosis) of Caco-2 cell cultures after a 15-min (not shown) or 30-min (Fig. 3B) treatment with 1 μg/ml CPE. For example, after a 30-min treatment with this CPE dose, the presence of GSK′840 or GSK′872 caused an 84.4% ± 4.4% or 83.3% ± 6.3% decrease, respectively, in annexin V staining. No necrotic cells were observed after this treatment with a low CPE concentration in the presence of the RIP3 inhibitors (Fig. 3B). However, when treated for 30 min with 1 μg/ml CPE in the presence of GSK′840 or GSK′872, 5.6% ± 1.5% or 6.3% ± 2.7%, respectively, of cells costained green and red, which is indicative of late-stage apoptosis, a situation where membrane permeability increases (26). Neither RIP3 inhibitor by itself caused staining with annexin V, and those cultures showed only very minimal red staining with EthD-III.

When Caco-2 cells were treated for 15 min (not shown) or 30 min (Fig. 3B) with 10 μg/ml CPE in the presence of 10 μM GSK′840 or GSK′872, apoptosis (annexin V staining) was absent. Furthermore, compared to cultures treated with this same high CPE dose in the absence of any RIP3 inhibitor, both inhibitors significantly (*P* ≤ 0.05) decreased the number of cells staining red with EthD-III after a 30-min treatment with 10 μg/ml CPE (Fig. 3B), i.e., after this treatment, the presence of GSK′840 or GSK′872 caused a 68.9% ± 23.7% or 71.0% ± 19.0% decrease, respectively, in red staining cells.

If, in the experiments shown in Fig. 3, GSK′840 and GSK′872 were protecting Caco-2 cells from CPE because RIP3 activation is a consequence of CPE pore formation, these RIP3 inhibitors should not affect CH-1 pore levels. To assess whether GSK′840 or GSK′872 affects CH-1 levels, Western blot analysis was performed with lysates from Caco-2 cells treated with 1 or 10 μg/ml CPE in the presence or absence of GSK′840 or GSK′872. The presence or absence of GSK′840 or GSK′872 did not affect CH-1 levels in Caco-2 cells treated for 30 (Fig. 3C) or 60 (not shown) min with either 1 or 10 μg/ml CPE.

### Effects of the MLKL oligomerization inhibitor NSA on CPE-induced Caco-2 cell death.

During the form of programmed necrosis known as necroptosis, activated RIP1 and RIP3 interact with other proteins to create the necrosome, which then induces MLKL to form a large (>250-kDa) MLKL octamer that serves as the effector for this cell death process (21, 22). Since RIP1 and RIP3 are involved in necrosis caused by a 10 μg/ml CPE treatment of Caco-2 cells (Fig. 2 and 3), experiments were performed with 2.5 μM necrosulfonamide (NSA), an MLKL oligomerization inhibitor (27, 33, 34), to begin evaluating if the form of necrosis caused by high CPE concentrations involves necroptosis.

**FIG 4:**
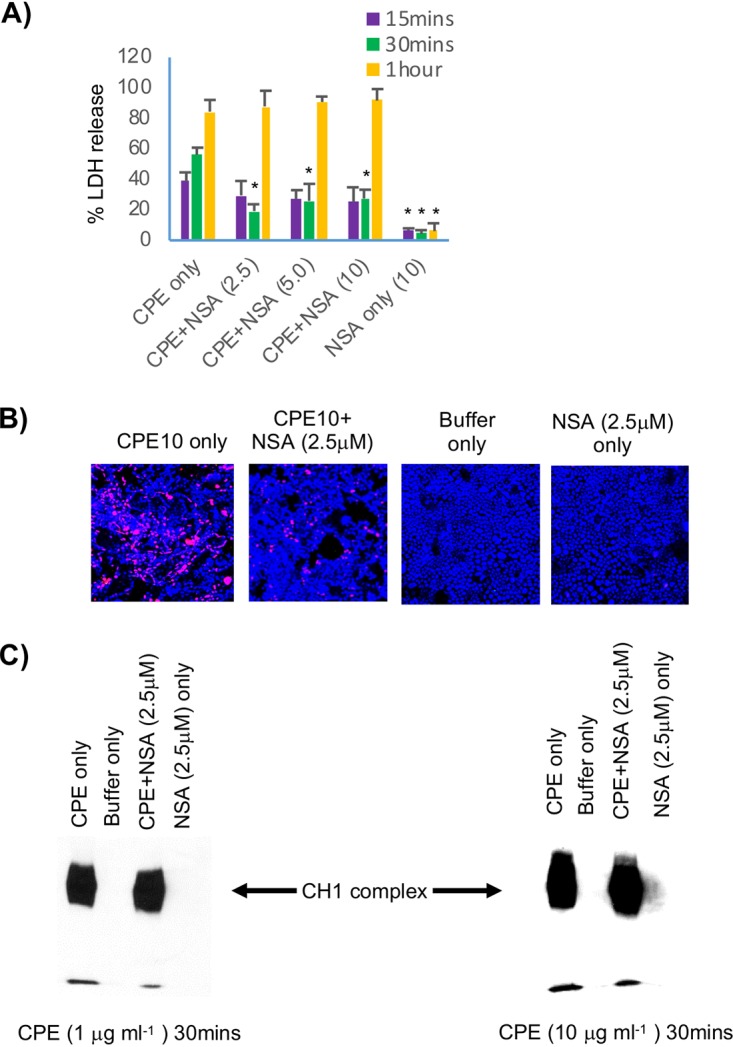
Effects of the MLKL oligomerization inhibitor NSA on CPE-induced cell death. (A) LDH release. Caco-2 cells were pretreated with HBSS that did or did not contain NSA at the indicated concentrations and then treated at 37°C for the indicated time with HBSS or HBSS containing 10 μg ml^−1^ of CPE, along with the same concentrations of NSA used, if any, for preincubation. LDH release was then assessed. Results shown are the means from 3 repetitions. Error bars represents the SEM. An asterisk indicates significantly different (*P* ≤ 0.05) values from CPE alone (no inhibitor). (B) Fluorescence microscopy. Caco-2 cells pretreated with HBSS that did or did not contain a 2.5 μM concentration of NSA were challenged for 30 min at 37°C with HBSS that did or did not contain 10 μg ml^−1^ CPE and (if used for pretreatment) 2.5 μM NSA. Treated cultures were then stained with an apoptotic/necrotic/healthy cell detection kit, where green cells are apoptotic (due to annexin V staining), red cells are necrotic (due to EthD-III staining), and total cells are blue (due to Hoechst 33342 staining). For each treatment, stained cells in at least 3 fields were quantified for each of 3 repetitions. Representative fields are shown. (C) Western blot analysis of CPE pore complex formation. Caco-2 cells pretreated for 2 h at 37°C in HBSS with or without 2.5 μM NSA then were treated for 30 min or 1 h (not shown) at 37°C with HBSS that contained 1 μg ml^−1^ (left) or 10 μg ml^−1^ (right) CPE plus the same concentration of NSA, if any, used for pretreatment. Cell lysates were subjected to CPE Western blotting (see Materials and Methods). Representative results from three repetitions are shown.

As shown in Fig. 4A, the presence of even a 2.5 μM concentration of NSA caused a significant 66.3% decrease in the LDH release induced by treatment of Caco-2 cells for 30 min with 10 μg/ml CPE. When these cultures were examined by fluorescence microscopy, the presence of 2.5 μM NSA significantly (*P* ≤ 0.05) reduced the necrosis caused by treatment with this high CPE concentration for 30 min, i.e., the number of necrotic, red-staining cells decreased by 71.5% ± 5.2% when 2.5 μM NSA was present during treatment with 10 μg/ml CPE (Fig. 4B). This effect was not due to NSA disrupting CH-1 pore formation, since CH-1 levels were not affected by the presence of NSA when Caco-2 cells were treated with this high CPE concentration (Fig. 4C).

As expected since MLKL oligomerization is not a feature of caspase-3 mediated apoptosis, the presence of NSA had no significant effect on either caspase-3 activation or the delayed LDH release caused by 1 μg/ml CPE (data not shown), and NSA alone (no CPE) did not cause LDH release or cell death staining (Fig. 4).

### Effects of RIP1, RIP3, or MLKL inhibitors on CPE-induced MLKL oligomerization in Caco-2 cells.

**FIG 5:**
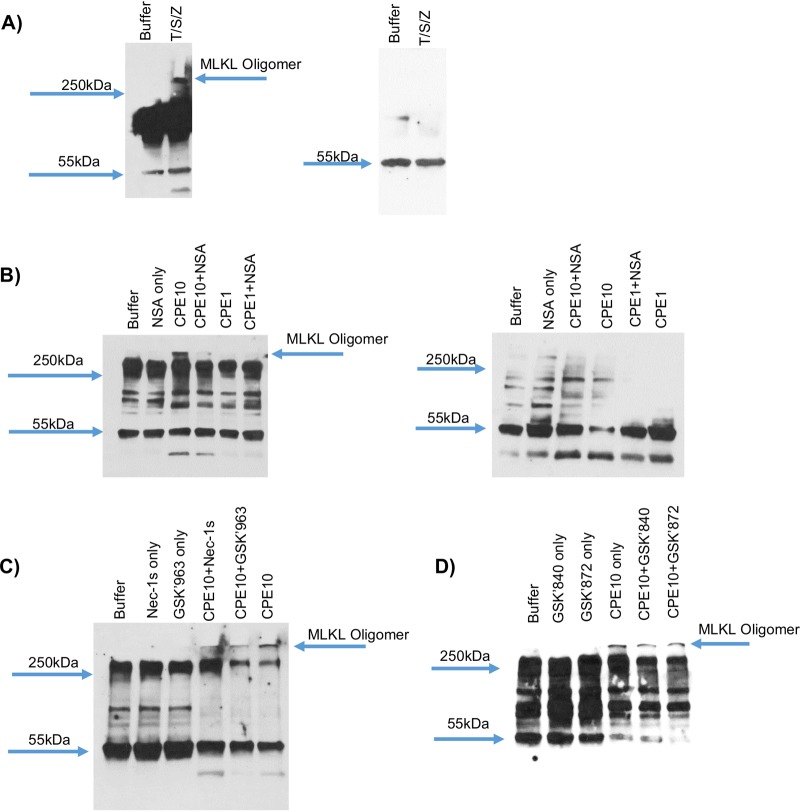
High CPE concentration induces MLKL oligomerization, which can be reduced by RIP1, RIP3, or MLKL inhibitor. (A) Caco-2 cells treated with agents that induce necroptosis form a large, >250-kDa MLKL octameric oligomer (arrow). Caco-2 cells were treated for 6 h at 37°C with HBSS that did (T/S/Z) or did not (buffer) contain TNF-α, z-VAD, and Smac, followed by mixing lysates of these cells with Laemmli buffer that did not (left) or did (right) contain beta-mercaptoethanol, prior to Western blotting for MLKL. Note the presence of the >250-kDa MLKL oligomer in the T/S/Z lane. (B) NSA effects on CPE-induced MLKL oligomerization. Caco-2 cells were pretreated for 2 h at 37°C with HBSS that did or did not contain a 2.5 μM concentration of the MLKL oligomerization inhibitor NSA and then challenged for 20 min at 37°C with HBSS that did not or did contain 1 μg ml^−1^ or 10 μg ml^−1^ CPE plus (if used for pretreatment) 2.5 μM NSA. Cell lysates were mixed with Laemmli buffer that did not (left) or did (right) contain beta-mercaptoethanol, and those samples then were subjected to MLKL Western blotting. Note that formation of the high-molecular-weight MLKL oligomer is sensitive to NSA and reducing conditions. (C) RIP1 inhibitor effects on CPE-induced MLKL oligomerization. Caco-2 cells were pretreated for 2 h at 37°C with HBSS that did or did not contain a 10 μM concentration of RIP1 inhibitor Nec-1s or GSK′963 and then challenged for 20 min at 37°C with HBSS that did not or did contain 10 μg ml^−1^ CPE plus the same RIP1 inhibitor, if any, used for pretreatment. Aliquots of each sample were electrophoresed under nonreducing conditions and Western blotted for MLKL. (D) RIP3 inhibitor effects on MLKL oligomerization. Caco-2 cells were pretreated for 2 h at 37°C with HBSS that did or did not contain a 10 μM concentration of RIP3 inhibitor GSK′840 or GSK′872 and then challenged for 20 min at 37°C with HBSS that did not or did contain 10 μg ml^−1^ CPE plus the RIP3 inhibitor (if used for pretreatment). Aliquots of each sample were then electrophoresed under nonreducing conditions and Western blotted for MLKL. Panels A to D show representative results from at least 3 repetitions.

If necroptosis is the form of necrosis induced by treatment of Caco-2 cells with high CPE concentrations, as possibly suggested by results shown in Fig. 4, those cells should contain a large >250-kDa MLKL octamer that is the critical effector for necroptosis and whose stability is sensitive to reducing conditions (21, 29, 35–38). Before assessing this possibility, a control experiment was performed to confirm that Caco-2 cells can undergo oligomerization to form this MLKL octamer when treated with factors known to induce necroptosis. MLKL Western blotting of gels electrophoresed under nonreducing conditions (Fig. 5A, left) detected formation of a large >250-kDa MLKL oligomer in Caco-2 cells treated with tumor necrosis factor alpha (TNF-α), Z-Vad, and Smac, which in combination are known to induce MLKL oligomerization and necroptosis in other cells (36, 39). Consistent with the literature (35), this >250-kDa MLKL octameric oligomer (36), and MLKL tetramer intermediates, dissociated if the same samples were electrophoresed under reducing conditions (Fig. 5A, right).

When similarly examined by MLKL Western blotting under nonreducing conditions, the presence of a >250-kDa MLKL species was detected in Caco-2 cells treated with 10 μg/ml CPE for 20 min (Fig. 5B, left). This large MLKL oligomer was notably absent from cultures of control Caco-2 cells or Caco-2 cells treated with 1 μg/ml CPE. In addition, the >250-kDa MLKL octamer, and MLKL tetramer intermediates, were disrupted if the same samples were analyzed by Western blotting using reducing electrophoresis conditions (Fig. 5B, right).

If treatment of Caco-2 cells with the 10 μg/ml CPE concentration causes necroptosis, NSA should reduce formation of the >250-kDa MLKL octameric oligomer in those cells. As shown in Fig. 5B (left), the presence of NSA significantly (*P* ≤ 0.05) reduced formation of that large MLKL oligomer by 67.2% ± 12.1%. Since RIP1 and RIP3 form the necrosome to promote MLKL oligomerization (22), it would also be expected that the RIP1 and RIP3 inhibitors used in studies shown in Fig. 2 and 3 should reduce >250-kDa MLKL oligomer levels in Caco-2 cells treated with 10 μg/ml CPE. This effect was observed, as shown in Fig. 5C and D. The RIP1 inhibitors Nec-1s and GSK′963 significantly (*P* ≤ 0.05) reduced levels of the >250-kDa MLKL oligomer by 56.0% ± 31.0% and 67.2% ± 21.2%, respectively, while the RIP3 inhibitors GSK′840 and GSK′872 significantly (*P* ≤ 0.05) reduced levels of this MLKL oligomer by 62.5% ± 8.0% and 75.8% ± 15.2%, respectively.

### Calpain activation is involved in CPE-induced necroptosis in Caco-2 cells.

**FIG 6:**
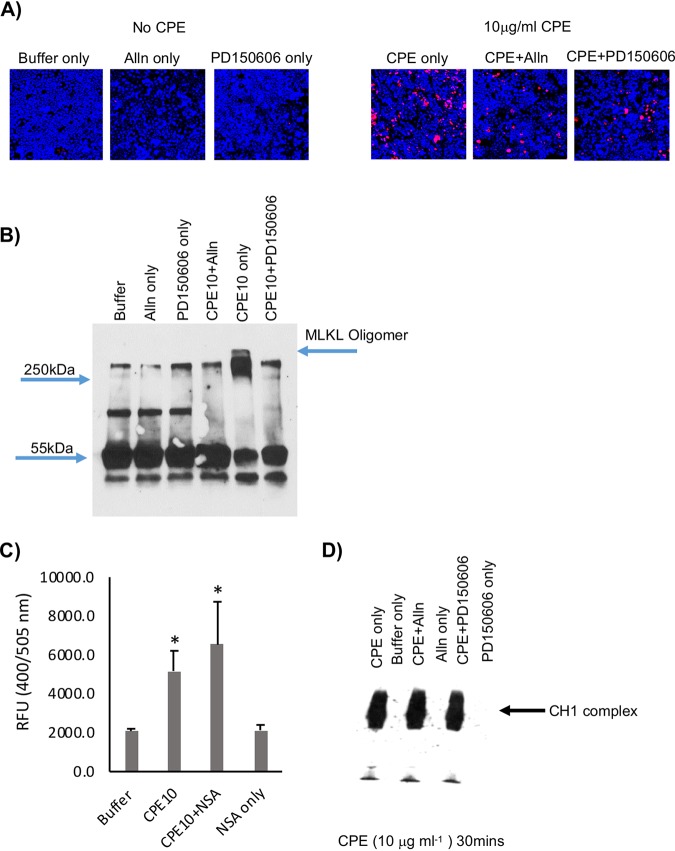
Effects of calpain inhibitors on necrosis and MLKL oligomerization induced by treatment with a high CPE concentration. (A) Fluorescence microscopy. Caco-2 cells were pretreated with HBSS that did or did not contain a 1 mM or 50 μM concentration, respectively, of calpain inhibitor ALLN or PD150606 and then challenged for 30 min at 37°C with HBSS that did not contain CPE (left) or contained 10 μg ml^−1^ (right) CPE and (if used for pretreatment) a 1 mM or 50 μM concentration, respectively, of ALLN or PD150606. Treated cultures then were stained with an apoptotic/necrotic/healthy cell detection kit, where green cells are apoptotic (due to annexin V staining), red cells are necrotic (due to EthD-III staining), and total cells are blue (due to Hoechst 33342 staining). For each treatment, stained cells in at least 3 fields were quantified for each of 3 repetitions. Representative fields are shown. (B) Calpain inhibitor effects on MLKL oligomerization. Caco-2 cells were pretreated with HBSS that did or did not contain a 1 mM or 50 μM concentration, respectively, of the calpain inhibitor ALLN or PD150606 and then challenged for 20 min at 37°C with HBSS that did not or did contain 10 μg ml^−1^ of CPE plus the same concentrations, if any, of ALLN or PD150606 used during pretreatment. Lysates of these cells were mixed with Laemmli buffer that contained beta-mercaptoethanol. Those samples were then subjected to MLKL Western blotting to detect the presence of the large (>250-kDa) MLKL octameric oligomer (arrow). Representative results from three repetitions are shown. (C) NSA effects on calpain activity in CPE-treated Caco-2 cells. Caco-2 cells preincubated with 2.5 μM NSA then were treated for 20 min at 37°C with HBSS that contained CPE (10 μg ml^−1^) and 2.5 μM NSA, if used for pretreatment. Control cells received HBSS or HBSS with 2.5 μM NSA, but no CPE, for pretreatment and treatment. Calpain activity in lysates of these cells was determined using the calpain activity kit (Abcam). Shown are the means from three experiments. Error bars represent the standard errors of the means. Significantly different (*P* ≤ 0.05) values from CPE alone (no inhibitor) are indicated by an asterisk. (D) Calpain inhibitor effects on CPE complex formation. Caco-2 cells pretreated with HBSS that did or did not contain a 1 mM or 50 μM concentration, respectively, of the calpain inhibitor ALLN or PD150606 were then challenged for 20 min at 37°C with HBSS that did not or did contain 10 μg ml^−1^ CPE plus the same calpain inhibitor (if used for pretreatment). Aliquots were then subjected to CPE Western blotting. A representative gel from three repetitions is shown.

Our previous study (17) reported that calpain activation is important for CPE-induced necrosis in Caco-2 cells, which was confirmed in the current study. As shown in Fig. 6A, the presence of two calpain inhibitors, i.e., ALLN and PD150606 (40, 41), significantly (*P* ≤ 0.05) reduced necrosis staining by 74.6% ± 6.0% and 70.0% ± 7.1%, respectively, after 30 min of treatment with 10 μg/ml CPE.

The activation of calpain in CPE-treated Caco-2 cells involves a CPE dose-dependent influx of Ca^2+^ (17). Some reports indicated that the MLKL octameric oligomer increases Ca^2+^ influx into host cells (39). Therefore, the two calpain inhibitors were used to discriminate whether, in Caco-2 cells treated with 10 μg/ml CPE, Ca^2+^ influx through the CPE pore triggers calpain activation to induce formation of the >250-kDa MLKL oligomer or, instead, calpain activation is primarily a consequence of Ca^2+^ influx due to MLKL oligomerization. As shown in Fig. 6B, the presence of either ALLN or PD150606 significantly (*P* ≤ 0.05) reduced levels of the high-molecular-weight MLKL oligomer present in Caco-2 cells treated with the high CPE concentration. Specifically, levels of the >250-kDa MLKL oligomer decreased by 86.5% ± 17.0% or 74.3% ± 26.7%, respectively, when ALLN or PD150606 was present during 20-min treatment of Caco-2 cells with 10 μg/ml CPE, implying that calpain activation is important for formation of the large MLKL oligomer under these treatment conditions.

This conclusion received important support when an experiment determined that the MLKL oligomerization inhibitor NSA does not significantly affect calpain activation in Caco-2 cells treated with 10 μg/ml CPE (Fig. 6C). Consistent with that observation, the presence of calpain inhibitors did not affect CH-1 pore formation in Caco-2 cells treated with 10 μg/ml CPE (Fig. 6D).

### CPE-induced cell death pathway activation in other mammalian cell lines.

Experiments then assessed whether CPE also induces concentration-dependent activation of apoptosis versus necroptosis in other cell lines. For this comparison, T84 cells (as a second human enterocyte-like cell line) or Vero cells (nonenterocyte Green monkey cells sometimes used in CPE research [42–44]) were treated for 30 min with 1 or 10 μg/ml CPE in the presence or absence of RIP1 or RIP3 inhibitors or NSA, followed by staining for cell death and evaluation by fluorescence microscopy.

**FIG 7:**
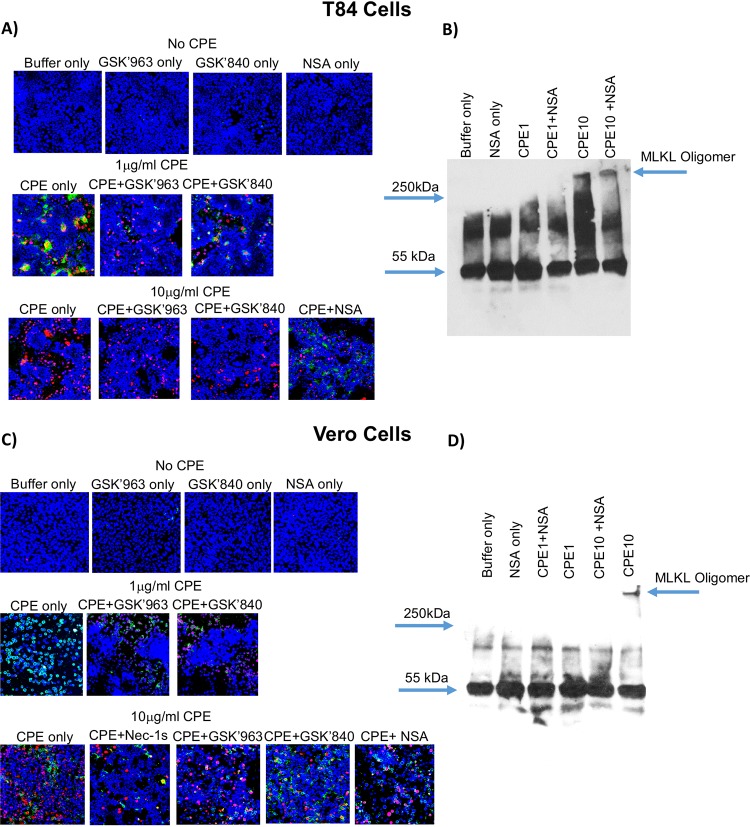
Effects of RIP1, RIP3, and MLKL inhibitors on CPE-induced cell death in T84 and Vero cells. (A and C) Fluorescence microscopy for CPE-induced cell death in T84 cells (A) or Vero cells (C) that were pretreated with HBSS that did or did not contain a 10 μM concentration of an RIP1 inhibitor (GSK'963 or Nec-1s, as indicated) or RIP3 inhibitor GSK′840 or a 2.5 μM concentration of the MLKL oligomerization inhibitor NSA and then challenged for 30 min at 37°C with HBSS that did not contain CPE (buffer, top row) or HBSS containing 1 μg ml^−1^ (middle row) or 10 μg ml^−1^ (bottom row) CPE and (if used for pretreatment) the same concentration of inhibitor used for pretreatment. Treated cultures were then stained with an apoptotic/necrotic/healthy cell detection kit, where green cells are apoptotic (due to annexin V staining), red cells are necrotic (due to EthD-III staining), and total cells are blue (due to Hoechst 33342 staining). For each treatment, stained cells in at least 3 fields were quantified for each of 3 repetitions. Representative fields are shown. (B and D) MLKL oligomerization in T84 cells or Vero cells. T84 (B) or Vero (D) cells were pretreated with HBSS that did or did not contain a 2.5 μM concentration of NSA and then challenged for 20 min at 37°C with HBSS that did not or did contain 1 or 10 μg ml^−1^ CPE plus the same concentration, if any, of NSA used during pretreatment. Lysates of these cells were mixed with Laemmli buffer that did not contain beta-mercaptoethanol. Those samples were then subjected to MLKL Western blotting to detect the presence of the large (>250-kDa) MLKL oligomer (arrow). Representative results from three repetitions are shown.

CPE treatment caused death pathway activation effects in T84 cells (Fig. 7A) similar to those described above for Caco-2 cells. After a 30-min treatment with 1 μg/ml CPE, T84 cells exhibited primarily apoptosis, i.e., 16.7% ± 3.5% of cells in these cultures were either apoptotic or late apoptotic, while 4.2% ± 0.3% of cells were necrotic (*P* ≤ 0.05). RIP1 and RIP3 were also involved in this apoptosis, since GSK′840 or GSK′963 significantly (*P* ≤ 0.05) reduced CPE-induced apoptosis/late apoptosis in these T84 cell cultures by 47.9% ± 11.7% or 63.5% ± 11.3%, respectively. Also similar to Caco-2 cells, necrosis (without apoptosis) was observed in T84 cell cultures treated with 10 μg/ml CPE, where 27.3% ± 1.6% of cells in those cultures developed necrosis. RIP1 and RIP3 were involved in this CPE-induced necrosis, since GSK′840 and GSK′963 significantly reduced (*P* ≤ 0.05) by 55.8% ± 16.5% or 43.8% ± 20%, respectively, the number of necrotic cells in T84 cell cultures treated with this high CPE concentration.

Cell death pathway activation in CPE-treated Vero cell cultures was generally similar, as identified, in the two enterocyte-like cell lines, although with a few differences (Fig. 7C). Vero cell cultures treated for 30 min with 1 μg/ml CPE exhibited predominantly apoptosis, with these cultures containing 39.3% ± 3.1% apoptotic cells versus 5.6% ± 1.2% necrotic cells. RIP1 and RIP3 were involved in this apoptosis, since GSK′840 or GSK′963 significantly (*P* ≤ 0.05) reduced, by 75.1 ± 9.6% or 56.6% ± 11.9%, respectively, the number of apoptotic cells in Vero cell cultures treated for 30 min with 1 μg/ml CPE. However, unlike enterocyte-like cell lines treated with this low CPE concentration in the presence of RIP1 or RIP3 inhibitors (Fig. 2, 3, and 7A), necrotic cell numbers in these Vero cell cultures increased by ∼2- or 3-fold, respectively. Vero cell cultures treated for 30 min with 10 μg/ml CPE developed predominantly necrosis but substantial apoptosis was also present, i.e., these cultures contained 41.7% ± 8.4% necrotic cells versus 17.9% ± 3.7% apoptotic cells. RIP3 is involved in this CPE-induced necrosis, since the presence of GSK′840 significantly (*P* ≤ 0.05) decreased, by 56.0% ± 12.3%, the number of necrotic cells present in Vero cell cultures treated with 10 μg/ml CPE. However, this reduction was off-set by a significant, nearly 2-fold increase in apoptotic cells. RIP1 was apparently involved in this necrosis, since GSK′963 reduced, by ∼35% ± 16.7%, the numbers of both apoptotic and necrotic cells present in Vero cell cultures treated for 30 min with 10 μg/ml CPE, although those protective effects did not reach statistical significance. Therefore, this treatment was repeated using Nec-1s, which provided significant (*P* ≤ 0.05) protection against both apoptosis and necrosis caused by this high CPE concentration. Specifically, the presence of Nec-1s caused a 73.9% ± 13.0% or 52.2% ± 14.0% reduction, respectively, in the apoptosis and necrosis caused by 10 μg/ml CPE.

Since necrosis was either exclusively or predominantly the form of cell death occurring in cultures of T84 and Vero cells treated for 30 min with 10 μg/ml CPE, the effects of 2.5 μM NSA were tested to explore if this necrosis involves necroptosis (Fig. 7A and C). NSA significantly (*P* ≤ 0.05) reduced death from necrosis by 60.3% ± 12.3% or 58.9% ± 18.2% in T84 or Vero cells, respectively, treated for 30 min with 10 μg/ml CPE. However, unlike Caco-2 cells, the presence of NSA during this CPE treatment also significantly increased apoptosis in both Vero and T84 cell cultures. For example, when treated for 30 min with the high CPE concentration, apoptosis increased from 0% in the absence of NSA to 10.3% of cells in the presence of NSA. NSA alone (no CPE) did not affect cell death staining for either Vero or T84 cells (not shown).

Those NSA results suggested that, as determined for Caco-2 cells, treatment of Vero or T84 cells with 10 μg/ml CPE causes necrosis that involves necroptosis. This conclusion was confirmed by MLKL Western blotting (Fig. 7B and D), which detected the presence of a large (>250-kDa) MLKL oligomer in T84 or Vero cell cultures treated for 20 min with 10 μg/ml, but not 1 μg/ml, CPE. As expected if this species is the large MLKL octameric oligomer, NSA significantly inhibited (*P* ≤ 0.05) its formation by 71.2% ± 27.7% or 92.7% ± 1.8%, respectively, in T84 or Vero cell cultures treated with 10 μg/ml CPE. The presence of NSA during CPE treatment did not affect CPE large complex formation levels in these two cell lines (data not shown).

## DISCUSSION

Pore-forming toxins (PFTs), the largest class of bacterial toxins, often play critical roles in bacterial virulence. Whereas it was once believed that PFTs kill host cells simply by inducing osmotic lysis, it has become apparent that these toxins can also use more subtle mechanisms to cause cell death. There has been some recent progress in understanding those nonlytic cell death pathways, e.g., for some PFTs, host cell proteins MLKL, RIP1, and RIP3 have now been implicated in the ability of PFTs to cause a programed form of necrosis known as necroptosis (45–47).

CPE is a PFT that induces multiple cell death mechanisms, i.e., in Caco-2 cells this toxin was previously shown to cause caspase 3-mediated apoptosis at low concentrations but necrosis at high concentrations (17, 18). Since apoptosis and necrosis sometimes involve RIP1 and RIP3 (30, 32, 48) and MLKL has been identified as the effector of necroptosis but not apoptosis (22), the current study had two initial goals. First, to evaluate whether RIP1, RIP3, and MLKL contribute to cell death caused by CPE and, second, to determine whether the form of necrosis induced using higher (but still pathophysiologic [19]) CPE concentrations involves necroptosis. Those initial questions were addressed using highly specific inhibitors of RIP1 or RIP3 kinase activity or MLKL oligomerization (23–25, 27, 28). The rationale for choosing this approach was that if an RIP1, RIP3, or MLKL inhibitor reduced cell death *in vitro*, that outcome might justify future *in vivo* testing of this inhibitor as a therapeutic agent against CPE-mediated illness. Although each RIP1 and RIP3 kinase inhibitor used in this study is highly specific, two inhibitors were tested for each kinase to further reduce possible off-target effects. A single MLKL inhibitor (NSA) was used, since, to our knowledge, this is the only commercially available MLKL oligomerization inhibitor; this was not a major limitation, since NSA effects can be assayed by electrophoresis to confirm directly that this inhibitor had affected MLKL oligomerization (29, 35–38).

Results using RIP1 and RIP3 inhibitors indicated that these two host kinases significantly contribute to CPE-induced Caco-2 cell death. To our knowledge, this study provides the first evidence that RIP1 and RIP3 can be involved in multiple cell death pathways induced by a single PFT, i.e., these two kinases contribute to both CPE-induced necrosis and apoptosis. While the RIP1 and RIP3 inhibitors provided significant protection against CPE-induced apoptosis and necrosis in Caco-2 cells, this protection was not complete and waned over time. The inability of RIP1 and RIP3 inhibitors to completely protect against CPE-induced Caco-2 cell death may be explainable by two nonexclusive possibilities. First, these inhibitors are not 100% effective, as shown in Fig. 5, where they did not completely block MLKL oligomerization caused by high CPE concentrations. Second, since results shown in Fig. 2 and 3 indicated that these inhibitors act after CPE pore formation, even in the presence of RIP1 or RIP3 inhibitors, CPE-treated Caco-2 cells may still be affected by other consequences of CPE pore formation, e.g., changes in intracellular concentrations of ions and precursors that can affect macromolecular synthesis (49).

The pathways of CPE-induced cell death identified for Caco-2 cells also generally occur in other cell lines, i.e., in T84 and Vero cells, low CPE concentrations also cause primarily apoptosis or late apoptosis while high CPE concentrations induce mainly necrosis. RIP1 and RIP3 inhibitors significantly reduced the apoptosis/late apoptosis caused by treatment with low CPE concentrations of either T84 or Vero cells. Those inhibitors also significantly reduced the necrosis caused by high CPE concentrations in those cells. Interestingly, unlike enterocyte-like Caco-2 or T84 cells, the presence of an RIP3 inhibitor shifted, from primarily necrosis to apoptosis, the cell death pathway activated in Vero cells treated with high CPE concentrations, indicating that there are some (unidentified) differences in CPE-induced activation of cell death pathways between different cell lines.

Several forms of necrosis are now recognized (21, 22). A major contribution of the current study is determining that the necrosis caused by high CPE concentrations involves the programmed form of necrosis known as necroptosis, i.e., this CPE-induced necrosis was shown to involve MLKL octameric oligomerization in Caco-2 cells, T84 cells, and Vero cells. That conclusion is consistent with some previous indirect findings. For example, CPE-induced necrosis was initially referred to as oncosis, because glycine, a membrane stabilizer, offered Caco-2 cells transient protection from high CPE dose-induced cell death (17, 18). More recent studies (46) demonstrated that glycine is similarly protective against the necroptosis caused by some other PFTs, such as pneumolysin. However, to our knowledge, CPE is the first PFT that has been specifically shown to cause caspase-3-mediated apoptosis at low concentrations but necroptosis at higher concentrations. Interestingly, other PFTs were recently reported to activate several caspases, but not caspase-3, during necroptosis (27); it should be evaluated whether, like CPE, low concentrations of other PFTs cause caspase-3-mediated apoptosis. Some differences exist between cell lines in their response to treatment with high CPE doses, since NSA significantly reduced necroptosis in both Caco-2 and T84 cells; however, there was a concomitant increase in apoptosis in those T84 cell cultures that was not seen for those Caco-2 cells. The basis for this shift in cell death pathway activation when T84 cells are treated with 10 μg/ml CPE in the presence of NSA requires further study.

Determining that different CPE concentrations induce apoptosis versus necroptosis in human enterocyte-like Caco-2 or T84 cells may have relevance for understanding type F disease. CPE concentrations associated with natural type F disease range from <1 to >100 μg/g feces, which overlaps the 1 or 10 μg/ml levels of CPE that trigger apoptosis or necroptosis, respectively, in human enterocyte-like Caco-2 or T84 cells. Given these substantial variations in CPE production among different type F strains during disease, it should be helpful for causing diarrhea and, thus, spore transmission, if both low and high CPE concentrations can cause intestinal cell death, even if by different mechanisms. CPE-induced activation of necroptosis versus apoptosis could also impact type F disease severity, but the cell death pathways induced by CPE in the intestines are not well understood. A recent study did show that 100 μg/ml CPE induces some caspase-3 activation in the mouse small intestine (50), but inhibiting this activation did not block CPE-induced intestinal pathology. However, inhibiting caspase-3 activation caused some reduction in pathology, although that effect did not reach statistical significance. Further *in vivo* studies are needed to establish whether caspase-3 activation and apoptosis are important during type F infections involving strains producing lower CPE concentrations or whether strains producing higher CPE concentrations cause intestinal necroptosis and if that effect facilitates lethal enterotoxemia.

Changes in homeostasis of cytoplasmic ions, including Ca^2+^, have been implicated in the necroptosis caused by other PFTs, e.g., pneumolysin (46). Ion dysregulation, particularly increased Ca^2+^ influx, is also involved in CPE-induced necroptosis, since our previous work (17, 18) showed that an influx of extracellular Ca^2+^, which increases calpain activity, is important for CPE-induced necrosis in Caco-2 cells. However, how cellular ion dysregulation mediates PFT-induced necroptosis has been unclear. One hypothesis is that changes in cytoplasmic ion concentrations directly affect RIP1, RIP3, or MLKL. Alternatively, ion dysregulation could induce the necroptosis pathway via some intermediate factor. If that second possibility is correct, then it would be important to identify this intermediate factor.

Therefore, perhaps the most significant findings of the current study are the NSA and calpain inhibitor results addressing whether an intermediate can contribute to necroptosis triggered by ion dysregulation. In CPE-treated Caco-2 cells, we previously established a link between levels of Ca^2+^ influx, calpain activation, and stimulation of specific cell death pathways, i.e., low CPE doses cause a limited Ca^2+^ influx that produces a mild increase in calpain activation to trigger cell death from caspase-3 mediated apoptosis, while higher CPE doses cause a massive Ca^2+^ influx that produces a strong calpain activation and induces necrotic death (17, 18). Coupling those findings with other studies linking MLKL oligomerization to increased cytoplasmic ion levels, possibly including Ca^2+^ (39, 46), it was conceivable that MLKL oligomerization during necroptosis is responsible for calpain activation in Caco-2 cells treated with high CPE concentrations. Alternatively, CPE pore formation might allow sufficient Ca^2+^ influx to activate calpain, which would then cause MLKL oligomerization and subsequent necroptosis. In this study, those possibilities were distinguished, and a key intermediate needed for MLKL oligomerization was identified by demonstrating that activated calpain is a major contributor to MLKL oligomerization in Caco-2 cells. While calpain activation has been linked to necroptosis caused by a few other non-toxin death stimuli (51, 52), these new CPE results represent, to our knowledge, the first linkage of calpain activation to PFT-induced necroptosis, as well as the first identification of an intermediate, i.e., calpain activation, for promoting MLKL oligomerization by any death stimuli. Whether calpain activation of MLKL oligomerization plays a central role as an intermediate in initiating necroptosis for other death stimuli, particularly for other PFTs, requires further investigation.

Currently, how MLKL oligomerization induces necroptosis is controversial. At least one previous study determined that MLKL can oligomerize into a cation-sensitive pore but, interestingly, that pore reportedly is not permeable to Ca^2+^ in the presence of Na^+^ and K^+^ (53). That finding is consistent with our current results indicating that the CPE pore, which forms prior to MLKL oligomerization, is primarily responsible for calcium-induced calpain activation in Caco-2 cells (and presumably other cells) treated with high CPE concentrations. However, it remains possible that MLKL oligomerization permits some supplemental Ca^2+^ influx beyond that allowed by the CPE pore.

**FIG 8:**
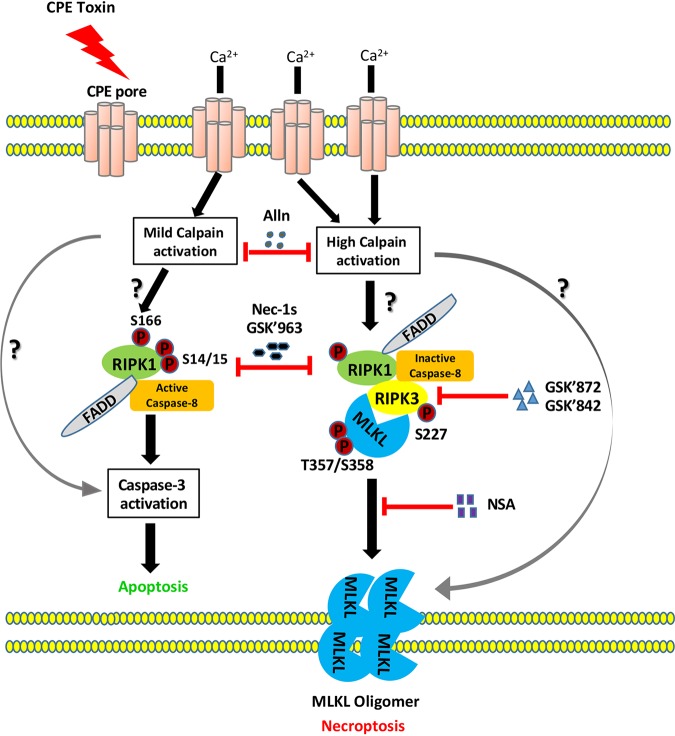
Updated model for CPE-mediated cell death. (Left pathway) When Caco-2 cells are treated with a low CPE concentration, modest numbers of CPE pores form to cause a limited Ca^2+^ influx that induces mild calpain activation. This limited calpain activation either directly or indirectly affects the complex containing RIP1 or activates caspase-3 to cause apoptosis. How RIP3 contributes to apoptosis remains unclear. (Right pathway) When Caco-2 cells are treated with a high CPE concentration, large numbers of CPE pores form to causes a strong Ca^2+^ influx that induces a massive calpain activation. This strong calpain activation either directly or indirectly affects the necrosome or acts postnecrosome formation to affect MLKL oligomerization. This effect then results in necroptosis.

Based upon the current and previous (18) findings, a working model for CPE-mediated necroptosis can be proposed (Fig. 8). Formation of many CPE pores permits a large Ca^2+^ influx that strongly activates calpain (17). Those high calpain activity levels then drive formation of the MLKL oligomer, which leads to further membrane destabilization by MLKL pore formation or another MLKL-mediated mechanism. This membrane disruption contributes to cell death by affecting metabolic processes such as macromolecular synthesis. Notably, this model remains incomplete. For example, future studies of CPE-induced cell death should identify the targets of activated calpain that promote MLKL oligomerization; is this MLKL itself or another intermediate? Also to be determined is how calpain activity differences mediate the triggering of CPE-induced apoptosis versus necroptosis.

## MATERIALS AND METHODS

### Materials.

As described previously, native Clostridium perfringens enterotoxin (CPE) was purified to homogeneity (54). Rabbit polyclonal antibody was raised against purified CPE, also as described previously (55). Sources and properties of antibodies used against RIP1, RIP3, and MLKL are described in Table 1. Protein concentrations of purified CPE or mammalian cell lysates were determined using the Pierce bicinchoninic acid protein assay kit (ThermoFisher).

**TABLE 1:** Sources and properties of antibodies used in this study

Antibody used^*a*^	Company	Catalog no.
RIP1 (D94C12)XP rabbit MAb*	Cell Signaling Technology	3493S
RIP1 anti-RIP clone 38/RIP (RUO) mouse MAb	BD BioSciences	610458
RIP3 (IMG-5846A) rabbit polyclonal*	Novus	NBP2-24588
RIP3 (E1Z1D) rabbit MAb*	Cell Signaling Technology	13526S
MLKL (D216N) rabbit MAb*	Cell Signaling Technology	14993S
MLKL rabbit polyclonal*	GeneTex	GTX107538

a *, Validated by supplier against appropriate knockout or knockdown cells. MAb, monoclonal antibody.

The RIP1 kinase activity inhibitor used in this study was Nec-1s (BioVision) or GSK′963 (Aobious), the RIP3 kinase inhibitor used was GSK′840 (Glixx Laboratories) or GSK′872 (EMD Millipore), the MLKL oligomerization inhibitor used was NSA (EMD Millipore), and calpain activity inhibitors used were ALLN (Cayman Chemicals) and PD150606 (Cayman Chemicals). All inhibitors were solubilized in dimethyl sulfoxide (DMSO) before use, with a final 0.1% concentration of DMSO present in cell cultures treated with inhibitors. Caco-2 cells treated with that DMSO concentration alone (no CPE or inhibitor) did not show any increased LDH release, caspase-3 activation, annexin V or ethidium homodimer III staining, or MLKL oligomerization compared to control Caco-2 cells (data not shown).

### Cell culture.

Authenticated Caco-2 cells and T84 cells were purchased from the ATCC. Authenticated Vero cells were kindly provided by William Goins. Caco-2 cells were cultured in 1× MEM (minimum essential medium) with Earle’s salts and l-glutamine (Corning, Cellgro) as well as 20% heat-inactivated fetal bovine serum (FBS; Sigma). Vero cells were cultured in DMEM (Dulbecco’s modification of Eagle’s medium) with 4.5 g/liter glucose, l-glutamine, and 5% FBS. T84 cells were grown in a 1:1 mixture of DMEM–F-12 medium with l-glutamine, HEPES, and 5% FBS. Cultures were grown to confluence in a humidified incubator at 37°C with 5% atmospheric CO_2_.

### RIP1, RIP3, and MLKL Western blot analysis.

Cells grown to confluence in 6-well plates were lysed with radioimmunoprecipitation assay buffer (RIPA; 25 mM Tris-HCl [pH 7.6], 150 mM NaCl, 1% NP-40, 1% sodium deoxycholate, 0.1% SDS [56]) containing protease inhibitor cocktail III (Research Products International) plus Benzonase (EMD Millipore). Lysates were collected and centrifuged to remove nonsoluble cell debris. Lysates were suspended in SDS-containing sample buffer, and samples (50 μg protein each) were then electrophoresed on a 10% polyacrylamide gel containing SDS. Separated proteins were transferred onto nitrocellulose membranes and blocked with 5% nonfat dry milk suspended in Tris-buffered saline with Tween 20 (TBS-T). The blocked membrane was probed with a RIP1, RIP3, or MLKL antibody (as specified in figure legends), each diluted 1:1,000 in 5% nonfat dry milk suspended in TBS-T. These blots then were reacted with secondary antibody conjugated to horseradish peroxidase (HRP; Sigma). Immunoreactive protein was visualized using SuperSignal West Pico chemiluminescence substrate (ThermoFisher).

### RIP1, RIP3, or MLKL inhibitor effects on CPE-induced release of LDH from Caco-2 cells.

Confluent Caco-2 cells cultured in 6-well plates were washed once with warm (37°C) Hanks’ balanced salt solution containing Ca^2+^ and Mg^2+^ (HBSS; Corning Cell Gro). Some of the washed cells then were pretreated with HBSS that did or did not contain an RIP1 inhibitor (Nec-1s or GSK′963) for 2 h at 37°C. The concentrations of Nec-1s used were 10, 50, or 100 μM (46), while the concentrations of GSK′963 used were 5, 10, or 20 μM (28). Similarly, some washed Caco-2 cell cultures were pretreated for 2 h at 37°C with HBSS that did or did not contain the RIP3 inhibitor GSK′840 or GSK′872. The concentrations of GSK′840 or GSK′872 used were 5, 10, or 20 μM (25). Finally, some cells were pretreated with HBSS that did or did not contain 2.5 μM NSA, an MLKL oligomerization inhibitor, for 2 h at 37°C.

Some pretreated cells then were washed twice with HBSS buffer before treatment for 15 min, 30 min, or 1 h at 37°C with HBSS containing CPE (1 or 10 μg ml^−1^), along with the same concentration of a RIP1, RIP3, or MLKL inhibitor, if any, used for pretreatment. Other pretreated Caco-2 cells were treated, as described above, with CPE-free HBSS that did or did not contain an inhibitor, as used during the pretreatment.

Following the treatments described above, cells were harvested and centrifuged at 2,000 × *g* for 5 min, and the supernatants were collected to measure LDH release from Caco-2 cells using an LDH release cytotoxicity detection kit (Roche). CPE-induced release of LDH was calculated according to the manufacturer’s instructions.

### RIP1, RIP3, and MLKL inhibitor effects on CPE-induced caspase-3/7 activity in Caco-2 cells.

Caco-2 cells cultured to confluence in 6-well plates were washed once with HBSS. The washed cells were then pretreated for 2 h at 37°C in HBSS or HBSS with a 10 μM concentration of an RIP1 (Nec-1s or GSK′963) or RIP3 (GSK′840 or GSK′872) inhibitor or a 2.5 μM concentration of the MLKL oligomerization inhibitor NSA. After two washes with HBSS, the pretreated cells were treated for 1 h at 37°C with HBSS containing CPE (1 or 10 μg ml^−1^) plus the same inhibitor, if any, used during pretreatment. Other pretreated cells were similarly treated with HBSS without CPE but containing the same RIP1, RIP3, or MLKL inhibitor, if any, present during the pretreatment.

Following their treatment, cells were gently collected by a rubber cell scraper and washed two times with HBSS buffer. The washed cells then were resuspended in lysis buffer containing 50 mM HEPES (pH 7.4), 0.1% 3-[(3-cholamidopropyl)-dimethylammonio]-1-propanesulfonate (CHAPS), 1 mM dithiothreitol, and 0.1 mM EDTA for 5 min on ice. Those lysates were centrifuged at 10,000 × *g* for 10 min at 4°C. Supernatants were tested for caspase-3/7 activity using 2 mM caspase-3/7 colorimetric substrate (Ac-DEVD-pNA [p-nitroaniline or pNA]; Sigma) dissolved in assay buffer (50 mM HEPES [pH 7.4], 100 mM NaCl, 0.1% CHAPS, 10 mM dithiothreitol, 1 mM EDTA, 10% glycerol). A 10-μl aliquot of each cell lysate (prepared as described above), 10 μl of caspase substrate solution, and assay buffer were combined in a 100-μl volume and incubated at 37°C for 4 h. Release of p-nitroaniline from the caspase-3 substrate was detected by measuring optical density at 405 nm (OD_405_) using a Bio-Rad plate reader. The amount of substrate cleaved was calculated according to the manufacturer’s instructions.

### CPE Western blot analysis.

Confluent Caco-2 cells in 6-well plates were washed once with HBSS and then pretreated for 2 h at 37°C in HBSS with a 10 μM concentration of an RIP1 or RIP3 inhibitor (Nec-1s, GSK′963, GSK′840, or GSK′872), a 2.5 μM concentration of the MLKL oligomerization inhibitor NSA, a 1 mM concentration of the calpain inhibitor ALLN, or a 50 μM concentration of the calpain inhibitor PD150606, as specified. The pretreated cells then were washed with HBSS and treated for 30 min or 1 h at 37°C with HBSS that did or did not contain CPE (1 or 10 μg ml^−1^) plus the same concentration of inhibitor, if any, used for pretreatment. In control wells, HBSS or HBSS with a 10 μM concentration of an RIP1, RIP3, MLKL, or calpain inhibitor but without CPE were used to pretreat and treat the Caco-2 cells. Similarly, confluent T84 or Vero cells in 6-well plates were washed once with HBSS and preincubated for 2 h at 37°C in HBSS without or with NSA (2.5 μM). The pretreated cells were then washed with HBSS and treated for 20 min at 37°C with HBSS or HBSS that contained CPE (1 or 10 μg ml^−1^) plus the same NSA concentration (2.5 μM), if any, used for pretreatment. In control wells, HBSS with NSA (2.5 μM), but no CPE, was used to pretreat and treat the T84 or Vero cells.

Following these treatments, cells were gently removed from each culture dish with a rubber cell scraper and collected by centrifugation at 2,000 × *g* for 5 min. The cell pellet was then resuspended in 50 μl of RIPA buffer with protease inhibitor cocktail III and Benzonase. Those samples were collected and 25 μg protein of each sample was mixed with Laemmli buffer and loaded onto an SDS-containing, 6% polyacrylamide gel. After electrophoresis, the separated proteins were electrotransferred onto a nitrocellulose membrane. The blots were blocked with 5% milk in Tris-buffered saline with 2% Tween 20 (TBS-T). Rabbit polyclonal anti-CPE antiserum (diluted 1:1,000 in TBS-T) was incubated with the blot overnight at 4°C. The blots were then washed three times with TBS-T and incubated for 1 h at room temperature (RT) with secondary goat polyclonal anti-rabbit IgG HRP-conjugated antibody (diluted 1:10,000 [Sigma]) suspended in 5% milk in TBS-T. Following three washes with TBS-T, SuperSignal West Pico substrate (ThermoFisher) was used to detect the CPE–CH-1 complex (16).

### Effect of inhibitors on MLKL oligomerization.

In a control experiment to demonstrate that Caco-2 cells can form the large >250-kDa MLKL oligomer when induced to undergo necroptosis, confluent Caco-2 cells cultured in 6-well plates were washed once with warm (37°C) HBSS. Some washed cells were treated for 6 h with HBSS that did or did not contain recombinant human TNF-α (10 ng/ml; Cayman Chemicals), Z-Vad (50 mM; R&D Systems), and Smac (100 nM; ChemieTek) to induce necroptosis (36, 37). These cells then were processed for MLKL Western blotting as described below.

To assess if CPE induces formation of a large MLKL oligomer, confluent Caco-2 cells cultured in 6-well plates were washed once with warm (37°C) HBSS (Corning Cell Gro). Some washed cells then were pretreated for 2 h at 37°C with HBSS that did or did not contain a calpain inhibitor (1 mM ALLN [14921] or 50 μM PD150606 [13859]; Cayman Chemicals), an RIP1 inhibitor (10 μM Nec-1s or GSK′963), an RIP3 inhibitor (10 μM of GSK′840 or GS′872), or 2.5 μM the MLKL oligomerization inhibitor NSA, followed by a 20-min challenge at 37°C with HBSS containing 10 μg/ml CPE plus the same concentration of the inhibitor, if any, used during pretreatment. Similarly, confluent T84 or Vero cells cultured in 6-well plates were washed with warm (37°C) HBSS and then pretreated with HBSS that did or did not contain NSA (2.5 μM) for 2 h at 37°C, followed by a 20-min challenge at 37°C with HBSS that did or did not contain a 1 or 10 μg/ml dose of CPE plus the same concentration of NSA, if any, used during pretreatment.

After these various treatments, cells were collected in precooled 1.5-ml Eppendorf tubes, centrifuged at 14,000 rpm for 5 min at 4°C, lysed with RIPA buffer with protease inhibitor cocktail III plus Benzonase for 20 min with rotation, and then centrifuged at 14,000 rpm for 20 min. Lysates were equalized for protein concentration, and samples (50 μg protein each) were boiled for 5 min with 2× SDS Laemmli buffer with or without beta-mercaptoethanol, as specified. The samples were cooled on ice, centrifuged for 5 min, and electrophoresed on a 4 to 20% gradient gel (Bio-Rad). After electrophoresis, the separated proteins were electrotransferred onto a nitrocellulose membrane. The blots were blocked with 5% milk in TBS-T, followed by incubation with MLKL antibody (1:1,000; 14993S; CST) overnight at 4°C. The blots then were washed three times with TBS-T and incubated for 1 h at RT with secondary goat polyclonal anti-rabbit IgG horseradish peroxidase-conjugated antibody (diluted 1:10,000 [Sigma]) suspended in 5% milk in TBS-T. Following three washes with TBS-T, Clarity Max Western ECL substrate (Bio-Rad) was used to detect the MLKL species.

### Fluorescence microscopy.

Caco-2 cells were cultured to confluence in an eight-chamber microscopy slide (Lab-Tek). The cells then were washed once with HBSS before pretreatment for 2 h at 37°C with HBSS that did or did not contain a 10 μM concentration of an RIP1 or RIP3 inhibitor (Nec-1s, GSK′963, GSK′840, or GSK′872), a 2.5 μM concentration of the MLKL oligomerization inhibitor NSA, a 1 mM concentration of the calpain inhibitor ALLN, or a 50 μM concentration of the calpain inhibitor PD150606. The pretreated cells were washed twice with HBSS buffer and then treated for 15 or 30 min at 37°C with HBSS that did or did not contain CPE (1 or 10 μg ml^−1^) plus the same concentration of inhibitor, if any, used for pretreatment. In control experiments, similarly pretreated cultures were treated with HBSS that did or did not contain the same concentration of one of these inhibitors but was CPE-free. Similarly, confluent T84 or Vero cells cultured in an eight-chamber microscopy slide were washed with warm (37°C) HBSS and then pretreated as specified with HBSS that did or did not contain 10 μM concentrations of Nec-1s, GSK′840, or GSK′963, or 2.5 μM NSA, for 1 h (Vero cells) or 2 h (T84 cells) at 37°C. After that pretreatment, cultures were challenged for 30 min at 37°C with HBSS that did or did not contain 1 or 10 μg/ml CPE plus the same concentration of inhibitor, if any, used during pretreatment.

Following these treatments, staining was performed using an apoptotic/necrotic/healthy cell detection kit (Promokine). The cells then were washed twice with 1× binding buffer (supplied in the kit). Staining solution was prepared by mixing 5 μl of fluorescein isothiocyanate (FITC)-annexin V, 5 μl of EthD-III, and 5 μl of Hoechst 33342 (each supplied in the kit) to 100 μl 1× binding buffer. The samples were incubated with the staining solution for 15 min at RT, protected from light. After the incubation, cells were washed once or twice with 1× binding buffer and covered with 1× binding buffer. Cells then were imaged using an Olympus confocal laser scanning biological microscope (Fluo View FV1000) with FV10-ASW (version 1.4) software. The excitation/emission wavelengths used were the following: for FITC-annexin V, 485/535 nm; for EthD-III, 595/613 nm (Texas Red); and for Hoechst 33342, 358/461 nm. Stained cells were quantified by ImageJ analysis (https://imagej.nih.gov) of 9 randomly chose microscope fields for each treatment condition, with each field containing ∼350 cells. The only exception was for cultures with substantial levels of late apoptosis, where cells were counted manually to ensure that the same cells were dual staining with both EthD-III and annexin-V. A sampling of fields from cells treated under other conditions were also counted manually, and the results obtained were always similar to ImageJ counts.

### Measurement of calpain activity.

Confluent Caco-2 cells in 6-well plates were washed once with HBSS and then preincubated for 2 h at 37°C in HBSS with 2.5 μM NSA. The pretreated cells were washed with HBSS and treated for 20 min at 37°C with HBSS that contained CPE (10 μg ml^−1^) and 2.5 μM NSA, if used for pretreatment. In control wells, HBSS or HBSS with 2.5 μM NSA, but no CPE, was used to pretreat and then treat the Caco-2 cells. Following these treatments, cells were gently removed from each culture dish with a rubber cell scraper and collected by centrifugation at 2,000 × *g* for 5 min. The cell pellet was then resuspended in 100 μl of extraction buffer provided in the calpain activity kit (Abcam), mixed several times by pipetting, and centrifuged at 14,000 rpm for 5 min at 4°C. Using 96-well black plates with a flat clear bottom, 200 μg protein of each sample was removed and adjusted to 85 μl/well with extraction buffer and 10 μl of 10× reaction buffer. Calpain substrate (5 μl) was added to each well, and plates were incubated at 37°C for 1 h protected from light. The output of the result was measured in a microplate reader that measured excitation/emission wavelengths of 400/505 nm.

### Densitometry.

Quantification of Western blot sample results was performed using ImageJ densitometric analysis. This analysis was performed on three separate Western blots for each experiment.

### Statistical analyses.

Statistical analyses were performed using GraphPad Prism software. One-way analysis of variance (ANOVA) using Dunnett’s multiple comparison was used to perform multiple-comparison tests.
